# Exploring the use of workplaces to recruit “hard-to-reach” male drinkers to a survey on alcohol use and awareness of health messages

**DOI:** 10.1186/s12889-021-10697-w

**Published:** 2021-03-31

**Authors:** Sarah Dance, Charlotte Dack, Celia Lasheras, Cathy McMahon, Paul Scott, Sally Adams

**Affiliations:** 1 Department of Psychology, University of Bath, Bath, BA2 7AY UK; 2 Public Health Department, Bath & North East Somerset Council, Bath, BA2 5RP UK

**Keywords:** Alcohol, Socioeconomic status, Recruitment, Hard-to-reach, Workplace, Survey

## Abstract

**Background:**

Lower socioeconomic status (SES) groups, particularly lower SES males, are at greater risk of alcohol-related harm than higher SES groups, despite drinking at the same level or less. However, they are rarely recruited for research through typical recruitment strategies. Consequently, limited evidence exists on patterns of alcohol use and effectiveness of public health messages for these groups. Using workplaces to recruit male drinkers from lower SES backgrounds may provide a feasible and accessible approach to research participation and enable improved understanding of alcohol use, drinking motives and acceptance of alcohol-related public health messages in this underrepresented and high-risk group. We investigated workplace-based strategies to recruit male drinkers from lower SES backgrounds. We also investigated their experiences and motivations for alcohol use, and acceptance of alcohol-related public health messages.

**Methods:**

A feasibility element investigated the effectiveness of workplace-based strategies to recruit male drinkers from lower SES backgrounds in the south west of England. A pilot element investigated this population’s experiences and motivations for alcohol use, and acceptance of alcohol-related public health messages, through a mixed-methods survey.

**Results:**

Feasibility results indicated that workplace-based recruitment strategies, including recruiting participants in person at their workplace and providing a financial incentive, effectively led to the recruitment of 84 male drinkers (70% recruitment rate), predominately from lower SES backgrounds, to a survey. Pilot results indicated that more than half of participants were at increasing risk of alcohol-related harm, and approximately one fifth engaged in weekly heavy episodic drinking. Participation in campaigns aimed at reducing alcohol use, and knowledge of government alcohol consumption guidelines, were low. Participants reported negative beliefs about alcohol including health effects, dependency and excess use, and financial and occupational effects. Positive beliefs about alcohol included relaxation, socialising, and enjoyment.

**Conclusions:**

Workplace-based recruitment, using in-person recruitment and a financial incentive, may be a feasible strategy to recruit male drinkers from lower SES backgrounds. Pilot results may direct larger scale research aiming to understand alcohol use in this population and inform targeted public health messages. Workplace-based recruitment may represent a promising avenue for future research aiming to tackle inequalities in participation in alcohol research.

## Background

Alcohol use is a leading risk factor for disease, disability and death globally. It is a causal factor in at least 200 disease and injury conditions [1], and results in three million deaths per year globally [2]. Socioeconomic status (SES) is also a significant factor in the association between alcohol use and risk of related harm [3]. Lower SES is associated with an increased risk of alcohol-attributable conditions, even after accounting for risk and confounding factors for these conditions [4]. An alcohol harm paradox exists, in which drinkers from lower SES groups are at greater risk of alcohol-related harm, despite drinking at the same level or less than drinkers from higher SES groups [3, 4]. Evidence from a systematic review, examining the role of alcohol use and drinking patterns in socioeconomic inequalities in mortality, highlights that a pattern of heavy episodic drinking among individuals of lower SES may partly explain the association between SES and mortality [5].

In the UK, those of lower SES are more likely to report heavy episodic drinking in the past week than those of higher SES [6]. Yet, those of lower SES also report drinking less frequently than those of higher SES; 47% of lower SES individuals in England reported drinking alcohol in the past week compared to 79% of higher SES individuals [7]. This alcohol harm paradox may have a greater impact in men than women. A systematic review examining gender differences in the distribution of alcohol-attributable mortality by SES indicates a three- to 10-fold higher risk of alcohol-attributable mortality in men of lower SES than women of lower SES [8]. Supporting evidence indicates that younger males who live in deprived neighbourhoods are most likely to engage in heavy episodic drinking, however males aged 35 to 64 years old showed the greatest increase in heavy episodic drinking in deprived neighbourhoods [6].

The disproportionate alcohol-related harm experienced by lower SES men requires investigation of this paradoxical relationship. However, lower SES individuals are under-represented in research investigating alcohol use, and in health research more generally, compared to higher SES individuals [9]. Consequently, they are often considered to be “hard-to-reach” in terms of recruitment to and participation in research studies [10]. Alternatively, this lack of representation may reflect that the researchers themselves are “hard-to-reach” due to their use of ineffective recruitment methods for this population, such as recruitment in university settings. Currently, researchers are not actively investigating how to recruit these participants through effective methods. Moreover, research may be limited by a lack of culturally appropriate information that is not relevant or tailored to the target population about participating in research [11], and the time commitment required to participate [12]. Developing research participation materials that are tailored to target populations may increase participation of under-represented populations in research studies [11]. At present, there is limited evidence on effective strategies to recruit lower SES groups, which highlights the need to explore the use of different recruitment methods for different populations. Therefore, researchers may need to explore the feasibility of strategies to recruit under-represented groups to enable their inclusion in research. By failing to include such groups in research, there is limited evidence on the motivations and experiences which may underlie the alcohol harm paradox, and on the effectiveness of alcohol-related public health messages in male drinkers from lower SES backgrounds. Understanding the experiences and motivations for drinking in this group may enable the development of targeted health messages for individuals with the highest burden of illness. Some evidence suggests that community-based recruitment strategies may be more effective at recruiting male drinkers from lower SES backgrounds than recruitment through identifying participants from primary care registers [13], which often fails to establish contact with these individuals to enable them to be invited to participate [14]. Workplaces may represent an accessible and feasible community-based location for recruitment [15]. Whilst recruitment through workplaces targets a working lower SES population, it may not capture all lower SES drinking males, such as those unemployed. However, those in routine and manual occupations experience high levels of alcohol-related harm [16–18]. Moreover, hazardous physical working conditions and low job control may contribute to health inequalities among working populations [19].

As male drinkers from lower SES backgrounds are at disproportionately high risk of alcohol-related harm [20], and are under-represented in research investigating alcohol use, this study had the following aims.

The feasibility aim was:

1. To explore whether workplace-based recruitment strategies are feasible for recruiting male drinkers from lower SES backgrounds

The pilot aims were:

2. To examine the experiences and motivations for alcohol use in males from lower SES backgrounds
3. To explore whether current alcohol-related public health messages are acceptable to male drinkers from lower SES backgrounds

## Methods

### Design

This study had two simultaneous elements. A feasibility element investigated the effectiveness of workplace-based strategies to recruit male drinkers from lower SES backgrounds to a survey. Recruitment strategies, including in person recruitment, employer email, employer noticeboard, and employer social media, were compared. Incentive strategies, including a survey with an information sheet informing of a prize draw, and a survey with an information sheet without this information, were compared. A pilot element investigated male drinker’s experiences and motivations of alcohol use, and acceptance of current alcohol-related health messages. A concurrent-nested, mixed-method design was used, in which a survey collected quantitative and qualitative data.

### Recruitment

#### Feasibility recruitment

**Fig. 1:**
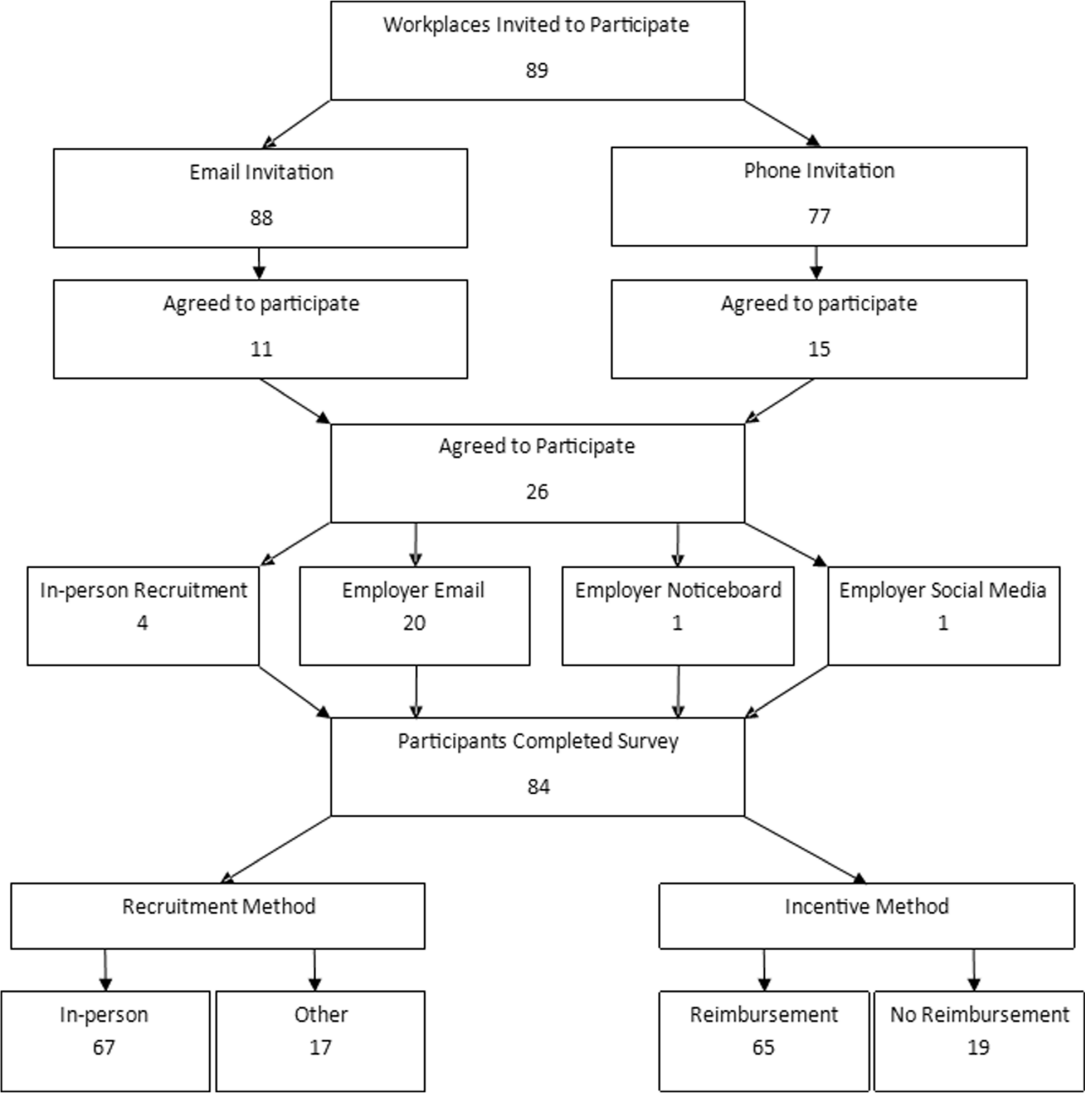
Recruitment strategy. A flow diagram of recruitment strategies for workplaces and participants

Participants were recruited through workplaces in a local authority in the south west of England, with a focus on workplaces mainly employing men working in routine and manual occupations. These workplaces were identified through working with the public health department of a local authority, and online searches for relevant organisations. A total of 89 workplaces, involving a combination of larger national and smaller independent organisations, were invited to take part. Eighty-eight were invited to participate through an email, of which 11 agreed. Those who did not respond to an email (77 workplaces) or had no email address (1 workplace) were invited to participate through a phone call, of which 15 agreed. The 26 workplaces which agreed to participate were asked which methods of recruitment they would be willing to take part in. We allowed workplaces to choose their recruitment strategy to increase uptake and cooperation, and to explore the acceptability of different recruitment strategies to workplaces. Four workplaces agreed to participate by allowing the researchers to recruit participants in person at their workplace, 20 workplaces agreed to participate by distributing a survey via their employer email, one workplace agreed to participate by distributing a survey via their employer noticeboard, and one workplace agreed to participate by distributing a survey via their employer social media. For the purpose of analyses, recruitment strategies were collapsed into two categories: in-person recruitment strategy and the other recruitment strategies combined (see Fig. 1).

#### Pilot recruitment

Ethical approval was gained from the Psychology Research Ethics Committee at the University of Bath before conducting the study (ethical approval code: 19–131). The study was conducted according to the revised declaration of Helsinki and Good Clinical Practice guidelines. To be eligible to participate, participants had to be male and aged 18 years old or above. They had to answer ‘yes’ to the question: ‘Do you drink alcohol?’. An a priori sample size was not calculated, due to the exploratory nature of participant recruitment. Eighty-four participants completed the survey (*M* age range = 45 to 54 years, *M* SES group = lower SES). Data collection took place between 17th June and 23rd July 2019.

### Measures

A survey about alcohol use and awareness of alcohol-related health messages was carried out with male drinkers. It took approximately five to 10 min to complete. Data were collected on participant demographics (including age and SES), alcohol use, location and social nature of alcohol use, awareness of current alcohol-related health messages, and beliefs about alcohol.

#### Age

Participants were asked to select their age, in years, using interval categories based on those used in the local authority for survey purposes: 18 to 34, 35 to 44, 45 to 54, 55 to 64, 65 to 74, 75 to 84, and 85+ [21].

#### SES

Participants were asked to select one category that best described their employment status from a list of job roles, based on nominal categories used by the United Kingdom’s Office for National Statistics (ONS): routine and manual occupations (eg: electricians and sales assistants); intermediate occupations (eg: secretaries and post office clerks); and higher managerial, administrative and professional occupations (eg: civil engineers and pharmacists) [22]. Each SES category contained a list of potential occupations with which the participant may identify. Categories of student/volunteering/apprentice and other were also included. Eighteen participants who selected “other”, and provided an optional job description, were categorised by occupational category according to the ONS (2010) guidelines, which provides examples of job roles for each SES category. Sixteen of these were categorised as lower SES, one was categorised as medium SES, and one was categorised as higher SES. Six participants selected “other” and did not provide a description of their job, and so were uncategorised by SES.

#### Alcohol use

Participants were asked to complete the Alcohol Use Disorders Identification Test-C questionnaire (AUDIT-C) [23]. This included three questions with responses on a five-point Likert scale. Questions asked participants about the frequency of alcohol use, the typical number of units consumed, and the frequency of heavy episodic drinking. This refers to drinking more than six units of alcohol for females and more than eight units of alcohol for males in a single session [24]. Prior to answering these questions, participants were shown an image displaying the units of alcohol in different alcoholic drinks, with the aim of improving the accuracy of reports of alcohol use. The total score of the questions provides a score of the risk of alcohol-related harm, with higher scores indicating greater risk of harm. Scores of zero to four indicate low risk of harm, scores of five to 10 indicate increasing risk of harm, and scores of 11 to 12 indicate high risk of harm.

#### Location and social nature of alcohol use

Participants were asked to select the location in which they normally drink alcohol, from the following responses: at home, out of the home (eg: pub, bar, restaurant, club), or both. Participants were also asked to select who they normally drank alcohol with, from the following responses: alone, with other people, or both.

#### Acceptance of alcohol-related public health messages

Participants were asked to estimate the government recommended maximum weekly alcohol consumption guideline. Ten responses, such as “0–2”, were rounded up to the highest value. Two responses stating “don’t know” were removed from the analysis.

Participants were also asked to select whether they had taken part in any campaigns or challenges aimed at cutting down or stopping drinking in the last 12 months, from the following responses: Yes, Dry January; Yes, Going Sober for October; Yes, other (free text response available); no; and don’t know. Participants could select more than one option.

#### Beliefs about alcohol

The following questions asked participants to report their beliefs about alcohol: “What do you think the positives of alcohol are?” and “What do you think the negatives of alcohol are?”. A free text response allowed them to report single or multiple beliefs, which were analysed using qualitative methods.

### Procedure

#### Feasibility procedure

Workplaces who agreed to participate through their email, noticeboard, or social media, were emailed a web link to the survey to distribute. Workplaces who agreed to participate through in person recruitment were contacted to arrange a convenient time to visit. Ten recruitment sessions were conducted in which the researchers visited sites across four workplaces to recruit participants. Workplaces were randomly assigned to receive one of two surveys: one including an information sheet detailing a prize draw opportunity upon survey completion, and one not detailing this in the information sheet. Workplaces were unaware of receiving different information sheets. This aimed to explore the impact of providing an incentive on recruitment rates (see Fig. 1).

#### Pilot procedure

The same researcher completed all in-person data collection, sometimes with the addition of another researcher. Participants who completed the survey in-person completed it via a tablet device provided by a researcher. Those who completed the survey through another recruitment method accessed a weblink to the survey from their employer, and completed it via their own electronic device. After reading the information sheet and being given an opportunity to ask any questions, participants were asked to give electronic consent by using survey tick boxes stating that they understood what participation involves and that they may withdraw at any time and up until the point of anonymisation. Participants then completed the survey and received a debrief sheet afterwards. The debrief sheet provided them with information about the aims and purposes of the study, and links to websites about alcohol consumption. It included a score of their risk of alcohol-related harm and brief advice on how to interpret this score, based on Public Health England’s (PHE) advice [25]. The debrief form informed all participants of the opportunity to enter a prize draw to win a £100 gift card by entering an email address, regardless of information sheet received.

### Analytic strategy

#### Feasibility analysis

In order to explore the feasibility of workplace-based recruitment strategies; recruitment, completion, and attrition rates for the survey were analysed using percentages. These rates were compared between the in-person recruitment strategy and the other recruitment strategies combined. This was due to the anonymous nature of survey, which prevented the research team knowing the recruitment method through which participants were recruited, except for the in-person recruitment strategy. Recruitment rate refers to all survey responses; including incomplete, complete, ineligible, and eligible survey responses. Completion rate refers to only complete and eligible survey responses. Attrition rate refers to incomplete or ineligible survey responses.

#### Pilot analysis

In order to explore the experiences and motivations of alcohol use, and acceptance of public health messages; quantitative survey data were analysed using descriptive analyses, including measures of central tendency. In order to make initial descriptive comparisons between the SES groups recruited in our sample, alcohol use was analysed between lower SES participants and higher SES participants using descriptive analyses. For the purposes of this comparison only, higher SES referred to both medium and high SES participants, and did not include those uncategorised by SES. IMB SPSS Statistics Version 25 was used to conduct descriptive analyses.

Qualitative survey data were analysed using content analysis, and an inductive approach was taken due to the lack of existing literature in this research area [26]. Consistent with an inductive approach, Erlingsson and Brysiewicz’s (2017) guidance on conducting conventional content analysis [27] was used to analyse the data. This includes developing familiarity with the data, separating the text into units of meaning and condensing these, generating codes, and creating categories.

## Results

### Feasibility results

Workplace participation by distributing a survey via the employers’ email was the most common way of taking part (76.92%), followed by allowing the researchers to recruit participants in person at their workplace (15.38%). Recruiting workplaces to participate through a phone call (16.85%) was more effective than recruiting workplaces through an email (12.36%) (see Fig. 1).

Eighty-four participants completed the survey out of 120 participants who were recruited, which is a 70% completion rate. An in-person recruitment strategy resulted in a higher completion rate and lower attrition rate (see Table 1).

**Table 1:** Feasibility of methods of recruiting participants

Measures of Feasibility	In-Person Recruitment Method	Other Recruitment Methods
Recruitment Rate	71 (59.16%)	49 (40.83%)
Completion Rate	67 (94.37%)	17 (34.69%)
Attrition Rate	4 (5.63%)	32 (65.31%)

The recruitment rate varied across in person recruitment sessions and ranged between five to 12 participants. The average recruitment rate per session was seven participants (see Table 2).

**Table 2:** Measures of feasibility of in-person recruitment method

Recruitment Sessions	Recruitment Rate	Attrition Rate	Completion Rate
Session 1	8	0	8
Session 2	6	0	6
Session 3	5	0	5
Session 4	6	0	6
Session 5	8	0	8
Session 6	5	0	5
Session 7	7	0	7
Session 8	12	2	10
Session 9	8	0	8
Session 10	6	2	4
Total	71	4	67
Mean Average	7	0	7

Recruitment session refers to a single time period of participant recruitment at a workplace

In order to explore the impact of providing an incentive on recruitment, participants either received an information sheet detailing a prize draw opportunity upon survey completion, or a survey which did not detail this in the information sheet. Although, all participants were informed that they could enter the prize draw during debrief. The survey with an information sheet informing of a prize draw opportunity resulted in a higher completion rate and lower attrition rate. Twelve participants entered the prize draw, which is an entrance rate of 14.29% (see Table 3).

**Table 3:** Feasibility of method of incentive for participants

Measures of Feasibility	Participants Initially Informed of Receiving Reimbursement	Participants Not Initially Informed of Receiving Reimbursement
Recruitment Rate	72 (60.00%)	48 (40.00%)
Completion Rate	65 (90.27%)	19 (39.58%)
Attrition Rate	7 (9.72%)	29 (60.42%)

### Pilot results

#### Participant demographics

Of those who completed the survey, the majority of participants (*n* = 51) were of lower SES (60.71%), 19 were of medium SES (22.62%), and eight were of higher SES (9.52%). Six were uncategorised by SES due to selecting “other” and not providing a job description.

Twenty-six participants were aged between 45 and 54 (30.95%), 22 were aged 18 to 34 (26.19%), 19 were aged 35 to 44 (22.61%), 15 were aged 55 to 64 (17.86%), and 2 were aged 65 to 74 (2.38%). None were aged 75 or above.

#### Risk of alcohol-related harm

Thirty-three participants were at low risk of harm (39.29%, *M* score = 2.75), 45 were at increasing risk of harm (53.57%, *M* score = 6.80), and 6 were at high risk of harm (7.14%, *M* score = 11.00). Therefore, 60.71% were at increasing risk or high risk of alcohol-related harm. Scores ranged between one and 11, and the mean score was in the increasing risk of harm category (*M* = 5.56, *SD* = 2.90).

#### Alcohol use

**Fig. 2:**
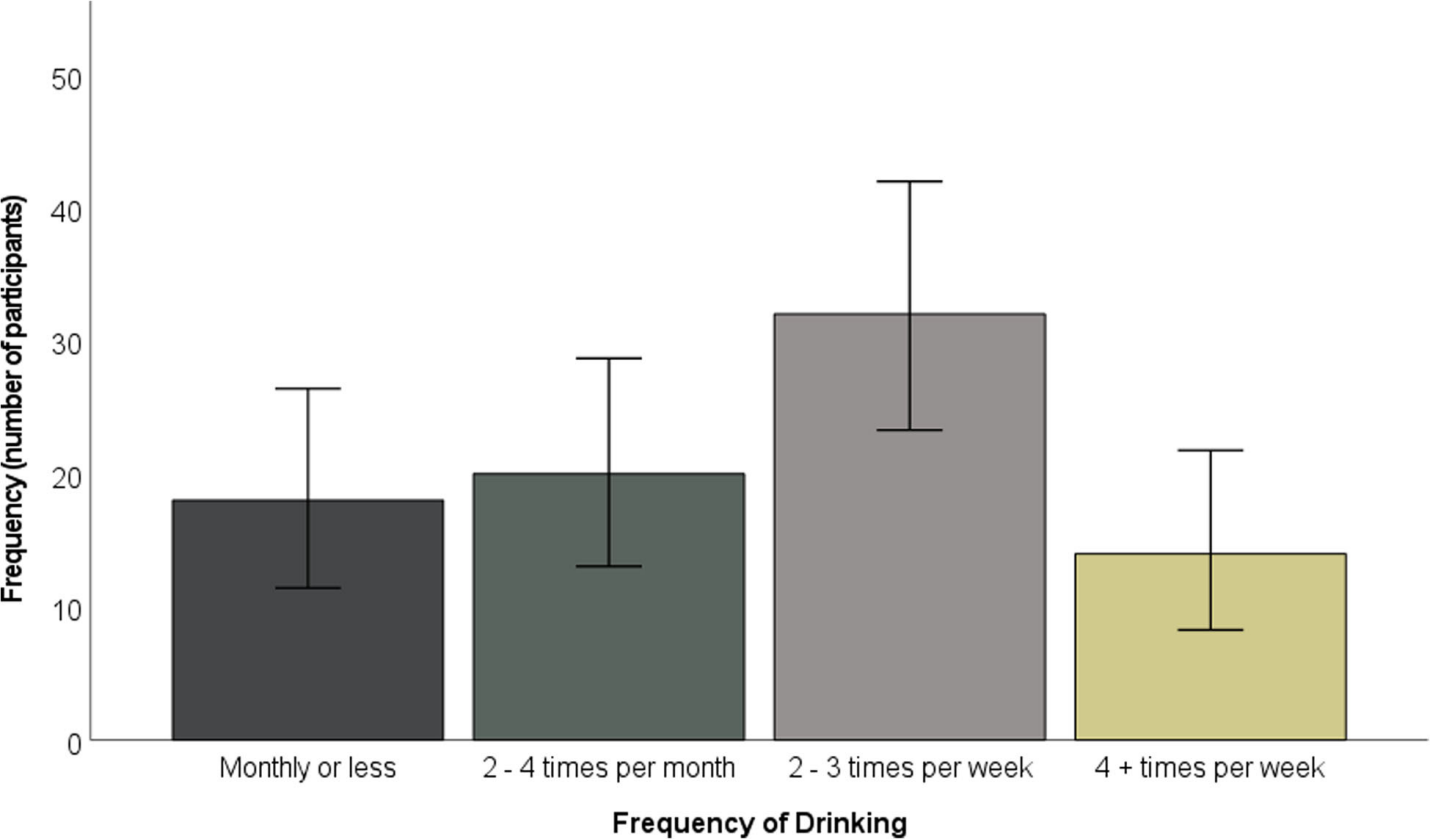
Frequency of drinking. Error bars represent 95% confidence intervals

**Fig. 3:**
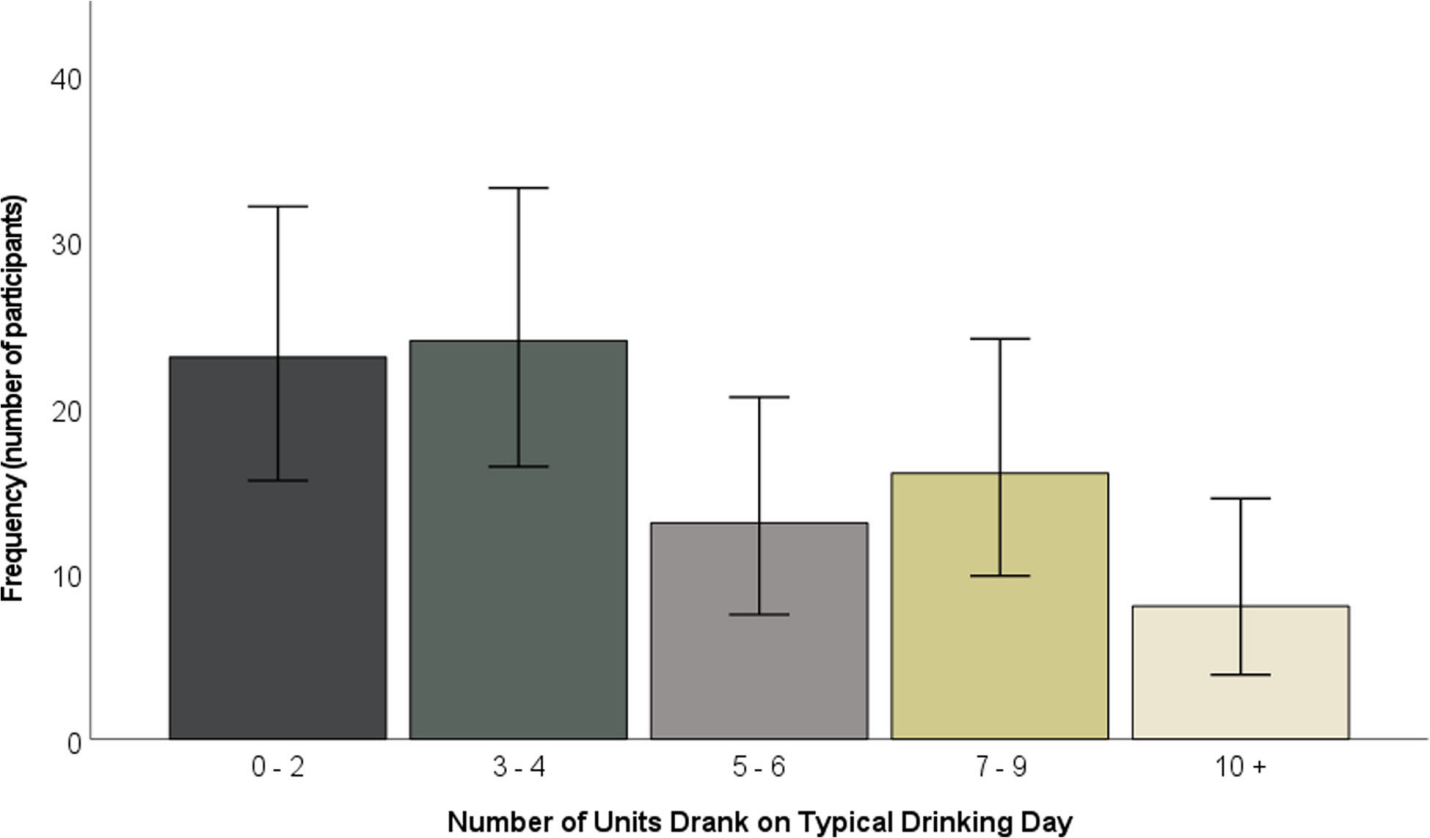
Number of units drank on typical drinking day. Error bars represent 95% confidence intervals

**Fig. 4:**
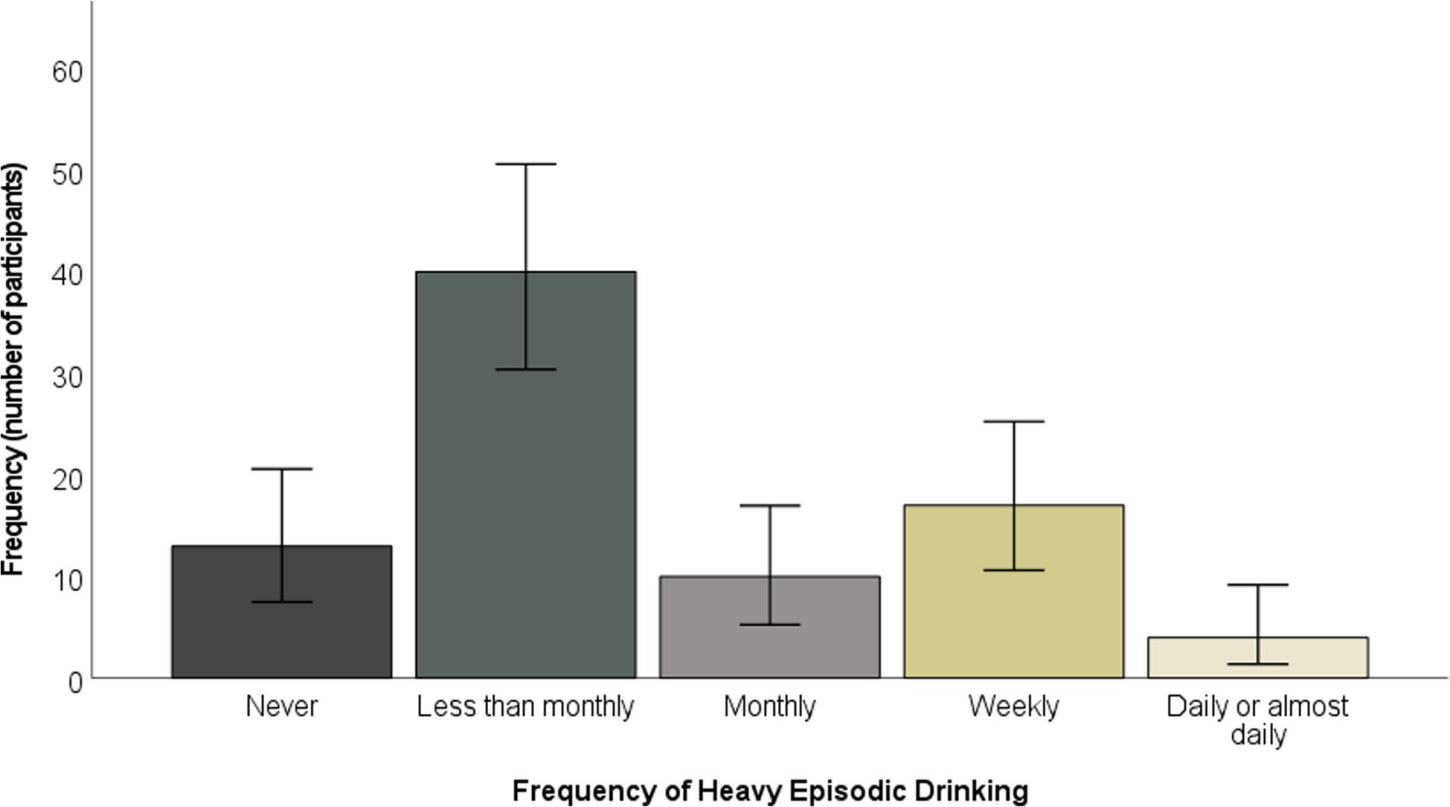
Frequency of heavy episodic drinking. Error bars represent 95% confidence intervals

The majority of participants drank alcohol two to three times per week (see Fig. 2). The majority typically drank three to 4 units, although over a quarter of participants (24 participants, 28.57%) typically drank at least seven units (see Fig. 3). Whilst just under half of participants engaged in heavy episodic drinking less than monthly, approximately one fifth (20.24%) engaged in weekly heavy episodic drinking (see Fig. 4).

#### Alcohol use across SES groups

**Fig. 5:**
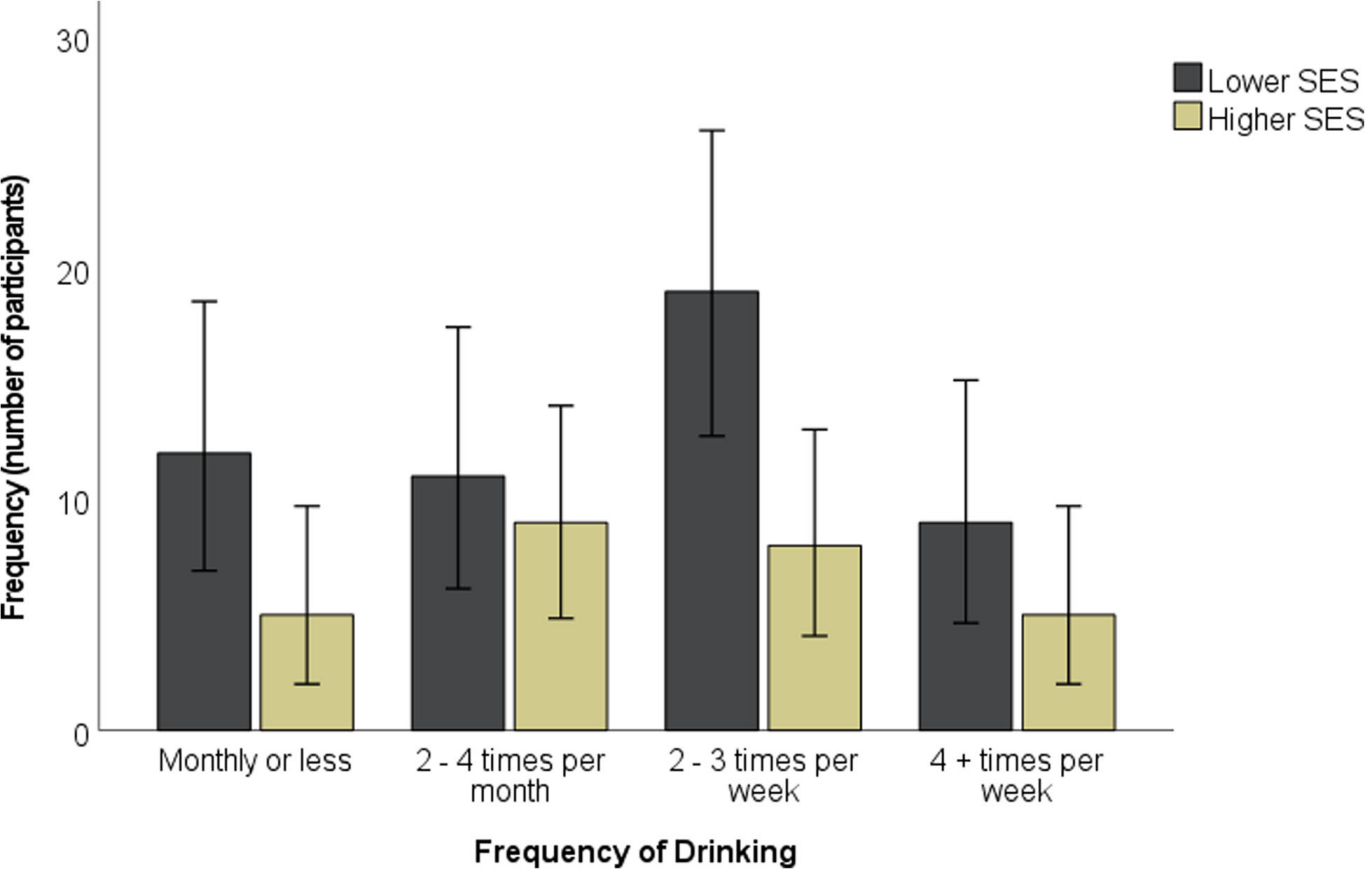
Comparison of alcohol use across lower and higher SES participants. Error bars represent 95% confidence intervals. This analysis only includes participants who were categorised by SES (*N* = 78). Higher SES refers to both medium and high SES participants (*N* = 27), and lower SES refers to low SES participants (*N* = 51)

The majority of lower SES participants drank alcohol two to three times per week, whereas the majority of higher SES participants (defined here as both medium and high SES participants) drank alcohol two to four times per month (see Fig. 5). The majority of both lower SES participants and higher SES participants typically drank 0 to 2 units on a typical drinking day, and engaged in heavy episodic drinking less than monthly.

#### Location and social nature of alcohol use

Drinking alcohol both at home and out of the home (36 participants, 42.86%) and solely out of the home (28 participants, 33.30%) was comparable. Fewer participants drank solely at home (20 participants, 23.81%).

Drinking alcohol with other people was the most common way to drink alcohol (56 participants, 66.60%). Fewer participants drank both alone and with others (25 participants, 29.8%) or only alone (three participants, 3.57%).

#### Acceptance of alcohol-related public health messages

Most participants (72 responses, 83.72%) had not taken part in a campaign or challenge aimed at reducing or stopping alcohol use, compared to those who had (14 responses, 16.28%). Of those who had taken part, seven had participated in another campaign (8.14%), six had participated in Dry January (6.98%), and one had participated in Going Sober for October (1.16%). “Other” campaigns or challenges participated in included “by myself”, “will power”, and “stopped for 3 months”.

Most participants underestimated the government alcohol consumption guidelines (74.39%). Fewer overestimated the guidelines (19.51%). The most common answer was eight units (18.29%), compared to five participants who correctly reported 14 units (6.10%).

#### Beliefs about alcohol

Eighty-four participants reported beliefs about alcohol, with some reporting multiple beliefs. Content analysis of negative beliefs about alcohol showed that the most common belief was that alcohol was linked to poor health outcomes, including liver disease and kidney damage (see Table 4). This was followed by beliefs that alcohol use could lead to dependency, such as addiction, and have negative impacts on work, finances, and safety. A small number of participants reported not being aware of any negative effects.

**Table 4:** Negative beliefs about alcohol

Belief	Examples	Frequency
Negative health effects	‘Liver failure’ ‘Kidney damage’	62
Excess and dependent use	‘Too much can be a bad thing’ ‘Addiction’	13
Financial and occupational effects	‘Losing money’ ‘Impact on work’	10
Safety risks	‘Violence’ ‘Engaging in dangerous activity’	10
Unknown or no negatives	‘Don’t know’ ‘None’	4
Conditional negatives	‘Up to each person’ ‘None if used sensibly’	2

Frequency refers to absolute numbers of beliefs

Content analysis of positive beliefs about alcohol showed that the most common beliefs were that alcohol facilitated relaxation (see Table 5). This was followed by beliefs about socialising, enjoyment, and improved characteristics. Participants also reported beliefs indicating that there are no positives of alcohol. A small number reported positive health effects.

**Table 5:** Positive beliefs about alcohol

Belief	Examples	Frequency
Relaxation	‘Chill time’ ‘Stress reliever’	30
Socialising	‘Social interactions’‘Social’	25
No positives	‘None’	16
Enjoyment	‘Fun’ ‘Enjoy the taste’	11
Improved characteristics	‘Dance better’ ‘Improve confidence’	9
Positive health effects	‘A healthier lifestyle’ ‘I think the odd glass of red wine is beneficial’	5

Frequency refers to absolute numbers of beliefs

## Discussion

This study explored the feasibility of workplace-based strategies to recruit male drinkers from lower SES backgrounds to a survey. The recruitment of 84 male drinkers from predominantly lower SES backgrounds indicates that workplace-based recruitment strategies, including in person recruitment and a financial incentive, are feasible to recruit drinkers from this population. This study also investigated the experiences and motivations for alcohol use, and acceptance of health messages, in this population. More than half of the participants were at increasing risk of alcohol-related harm and approximately one fifth engaged in weekly heavy episodic drinking, which is higher than rates reported in the UK general population [7, 28, 29]. Participation in campaigns aimed at reducing alcohol use, and accurate awareness of government alcohol consumption guidelines, were low. Health effects were the most common negative belief about drinking, and relaxation was the most common positive belief.

The feasibility findings extend the evidence base on the feasibility of methods to recruit male drinkers from lower SES backgrounds, and under-represented groups more generally. Previous evidence suggests that community-based recruitment strategies may be more effective at recruiting males from lower SES backgrounds than strategies such as primary care register recruitment [13]. Our findings suggest that recruitment through workplaces may be an effective community-based recruitment strategy for this group. The feasibility of this method may be related to information provided to participants that the research was not driven by the employer, which may have increased participants willingness to participate in the research. The effectiveness may also have been influenced by the presence of the researchers, as participants could ask questions about the research [30]. This recruitment strategy may be a useful alternative to those which have typically been less effective in recruiting perceived “hard-to-reach” populations, such as through primary care registers which often fails to make contact with such populations [13]. It may be more likely to reach lower SES males who would not respond to recruitment through medical services. Recruitment was also facilitated by accessing workplaces through working with a local authority public health department. As the employers were the gatekeepers to the target population, it was important to explore how to best recruit the employers to take part.

In addition, higher recruitment to a survey with an incentive is consistent with previous findings [31, 32]. This may be explained through social action theory [33], in which survey completion depends on the associated rewards, costs, and trust. The recruitment rate may be due to perceived high rewards of a prize draw. Yet, the low uptake of the incentive may be due to reduced trust associated with entering an email address after answering sensitive questions. The delayed nature of the prize draw may also play a role. Surveys with delayed notification of prize draw results have lower response rates than those with immediate notice due to the immediacy effect, in which individuals tend to choose immediate rewards over delayed ones [34]. Alternatively, the incentive used (a gift card) may have low acceptability in this population.

The pilot findings extend the evidence on alcohol use in lower socioeconomic status populations, which may improve understanding of the alcohol harm paradox. The rates of lower SES men at increasing risk (53.57%) and high risk (7.14%) of alcohol-related harm is higher than that reported for all men in the UK (24% and 3% respectively). Similarly, the rates of lower SES men at lower risk (39.29%) is lower than that reported for all men in the UK (72%) [28]. However, these comparisons are limited by methodological differences between the surveys and the sample sizes. Yet, they provide preliminary indications of alcohol use in lower SES men in relation to the general population. In our sample, greater numbers of lower SES participants reported drinking alcohol at each measure of frequency compared to higher SES participants. This contrasts with findings that 10% of higher SES individuals in Great Britain drank on at least five days in the week prior to interview in 2017, compared to 7% of lower SES individuals [29]. However, comparison across SES groups within our sample is limited by the greater number of lower SES participants recruited.

The higher rate of alcohol-related harm may be explained through differences in drinking patterns, as a higher rate of lower SES men had engaged in heavy episodic drinking on a weekly basis (20.24%) than individuals in the UK (16%) [29]; (15%) [7]. This is consistent with evidence highlighting an increased risk of heavy episodic drinking in men from lower SES backgrounds [6, 18, 35]. Moreover, it is also consistent with a systematic review examining the role of alcohol use and drinking patterns in socioeconomic inequalities in mortality that identified 15 to 30% of socioeconomic inequalities in mortality as being explained by heavy episodic drinking, compared to 5 to 15% of mortality as explained by total alcohol consumption [5]. Therefore, a pattern of heavy episodic drinking may explain the increased risk of alcohol-related harm in this population [3]. Heavy episodic drinking may increase the risk of alcohol-related harm due to removing the protective effects of moderate consumption for ischemic heart disease [36], stroke [37] and unintentional and intentional injuries [38]. Alternatively, the location in which participants were recruited may be a confounding factor, due to the relatively high rate of heavy episodic drinking in the south west of England (15%) compared to other areas in England, such as the south east (11%) [29]. The south west is also the region of England with the lowest proportion of non-drinkers (12%) compared to the highest proportion in the west Midlands (25%) [39]. Occupation may be another confounding factor, as workplace social norms about drinking predicts alcohol use amongst employees [40].

The beliefs about alcohol use identified may motivate alcohol use, as attitudes towards alcohol correlate with alcohol use intentions [41]. In terms of beliefs about relaxation, heavy episodic drinking occurs more frequently when experiencing stress, with a stronger effect of stress on heavy episodic drinking among lower SES individuals [42]. Men from lower SES backgrounds may be more likely to engage in heavy episodic drinking in order reduce stress and increase relaxation. This supports a model in which stress motivates alcohol use in those who use avoidant coping [43]. Therefore, relaxation may motivate alcohol use through a mechanism of coping. High rates of negative beliefs about health effects were reported despite comparatively higher rates of alcohol use than the general population. This supports evidence that knowledge of health effects alone does not affect behaviour change [44]. Findings of low participation in campaigns aimed at reducing alcohol consumption and awareness of alcohol consumption guidelines is similarly low in the general population [28, 45, 46]. However, it is not clear whether the underlying reasons are similar across SES groups, or whether this population may face unique barriers to increased participation in and awareness of public health messages.

Efforts to tackle alcohol-related inequalities could be informed by the development of targeted alcohol-related public health messages addressing the experiences and motivations identified in this high-risk population, such as a behavioural pattern of heavy episodic drinking. This supports recommendations for the development of public health strategies addressing alcohol-related inequalities by targeting patterns of heavy episodic drinking, rather than general alcohol consumption, among individuals from lower SES backgrounds [5]. However, substantial variation in drinking patterns within this population exists, with just under half of participants engaging in heavy episodic drinking on a less than monthly basis. Therefore, public health messages aiming to tackle health inequalities may also need to account for variation in drinking behaviours within this population. This suggests that whilst a pattern of heavy episodic drinking may partly explain the alcohol harm paradox, other factors may also have a role in explaining the increased alcohol-related harm observed in this population.

To our knowledge, this is the first exploration of the feasibility of workplace-based recruitment strategies to recruit male drinkers from lower SES backgrounds to a survey. This study identifies feasible methods to improve the inclusion of lower SES males in research aiming to tackle inequalities in alcohol use. Yet, limitations exist. The method used to recruit workplaces resulted in a relatively low uptake of organisations to the study; only 26 workplaces agreed to take part out of 89 workplaces who were invited to participate. Whilst response rate is an important initial outcome indicating which recruitment strategies are effective, it is unclear why some workplaces and participants chose not to participate. This may be improved by recruiting workplaces face-to-face and working with organisations overtime to build relationships. Maintaining flexibility and persistence in recruitment may be key to identifying the most effective methods of recruiting the gatekeepers to enable access to the target population [15]. The present sample may not be a representative sample of the target population given that some medium and higher SES participants were recruited in addition to lower SES participants. However, targeted sampling may not currently be feasible for recruiting lower SES working males due to relatively limited access to the population. Therefore, the recruitment strategy may require adaptation to improve the representativeness of the sample, eg: by recruiting across a larger geographical area and sample, or using methods such as snowball sampling and derived rapport. It is likely that a combination of strategies may be the best approach [47]. Additionally, the selection of participant recruitment strategies by workplaces may have introduced selection bias. However, given the lack of evidence available on effective strategies to recruit this population, it was necessary to explore which strategies were feasible to recruit participants from this population. It is possible that fewer workplaces would have participated if we had randomly assigned them to a recruitment strategy. Similarly, whilst the recruitment strategy was costly in terms of time spent recruiting workplaces and participants, it is possible that fewer participants from the target population would have been recruited without exploring how best to recruit this population.

The use of workplace-based recruitment prevented recruitment of male drinkers from lower SES backgrounds who are not working due to alcohol use disorder, unemployment, or not being of working age. This potentially limits the generalisability of our findings to all groups of lower SES males as occupation is only one of many indicators of SES [48]. Although, this study aimed to target those in routine and manual occupations due to their high levels of alcohol-related harm [16–18]. Additionally, the impact of the location that the survey was completed in on the accuracy of responses is unclear. Whilst participant confidentiality was ensured, and accuracy of responses was aimed to be improved through the use of an image displaying the units of alcohol in different drinks; it is possible that participants recruited in person may be less likely to accurately report their alcohol use at their workplace, such as those with safety critical roles in which high alcohol use could have serious consequences.

Future research may wish to explore the feasibility of workplace-based recruitment for this population across different geographical locations. Future studies could also explore the acceptability of different types of incentives, including immediate versus delayed; and the feasibility of workplace-based recruitment for other under-represented groups in research. The pilot findings also provide directions for future research aiming to better understand alcohol use in this population and to inform the development of targeted health messages about alcohol. Future studies could conduct larger scale research to build upon these initial pilot results. The prevalence of heavy episodic drinking as a possible mechanism involved in the high alcohol-related harm seen in male drinkers from lower SES backgrounds would be useful to explore across a larger sample. It may be useful to explore what barriers and facilitators affect participation in campaigns aiming to reduce alcohol consumption in this population. This may be best explored through qualitative methods.

Workplace-based recruitment, with the use of an in-person recruitment strategy and a financial incentive, may be a promising strategy for future research aiming to tackle inequalities in participation in alcohol research. Improved understanding of the experiences and motivations of alcohol use in male drinkers from lower SES backgrounds may advance knowledge of the mechanisms of the alcohol harm paradox and inform the development of targeted alcohol-related public health messages.

## Data Availability

The datasets used and/or analysed during the current study are available from the corresponding author on reasonable request.

## Abbreviations

SES: Refers to socioeconomic status
ONS: Refers to Office for National Statistics
AUDIT-C: Refers to Alcohol Use Disorders Identification Test-C questionnaire
PHE: Refers to Public Health England

## References

[1] World Health Organisation. Global status report on alcohol and health 2014. 2014. Available from: https://www.who.int/substance_abuse/publications/alcohol_2014/en/.

[2] World Health Organisation. Global status report on alcohol and health 2018. 2018. Available from: https://www.who.int/publications/i/item/9789241565639.

[3] Bellis MA, Hughes K, Nicholls J, Sheron N, Gilmore I, Jones L (2016). The alcohol harm paradox: using a national survey to explore how alcohol may disproportionately impact health in deprived individuals. BMC Public Health.

[4] Jones L, Bates G, McCoy E, Bellis MA (2015). Relationship between alcohol-attributable disease and socioeconomic status, and the role of alcohol consumption in this relationship: a systematic review and meta-analysis. BMC Public Health.

[5] Probst C, Kilian C, Sanchez S, Lange S, Rehm J (2020). The role of alcohol use and drinking patterns in socioeconomic inequalities in mortality: a systematic review. Lancet Public Health.

[6] Fone DL, Farewell DM, White J, Lyons RA, Dunstan FD (2013). Socioeconomic patterning of excess alcohol consumption and binge drinking: a cross-sectional study of multilevel associations with neighbourhood deprivation. BMJ Open.

[7] National Health Service. Statistics on Alcohol. England, 2018. 2018 [Available from: https://files.digital.nhs.uk/AD/C3036E/alc-eng-2018-rep.pdf.

[8] Probst C, Roerecke M, Behrendt S, Rehm J (2015). Gender differences in socioeconomic inequality of alcohol-attributable mortality: a systematic review and meta-analysis. Drug Alcohol Rev.

[9] Lorant V, Demarest S, Miermans P-J, Van Oyen H (2007). Survey error in measuring socio-economic risk factors of health status: a comparison of a survey and a census. Int J Epidemiol.

[10] Bonevski B, Randell M, Paul C, Chapman K, Twyman L, Bryant J, Brozek I, Hughes C (2014). Reaching the hard-to-reach: a systematic review of strategies for improving health and medical research with socially disadvantaged groups. BMC Med Res Methodol.

[11] Ford JG, Howerton MW, Lai GY, Gary TL, Bolen S, Gibbons MC, Tilburt J, Baffi C, Tanpitukpongse TP, Wilson RF, Powe NR, Bass EB (2008). Barriers to recruiting underrepresented populations to cancer clinical trials: a systematic review. Cancer..

[12] Paskett ED, Reeves KW, McLaughlin JM, Katz ML, McAlearney AS, Ruffin MT (2008). Recruitment of minority and underserved populations in the United States: the centers for population health and health disparities experience. Contemp Clin Trials.

[13] Crombie IK, Falconer DW, Irvine L, Norrie J, Williams B, Slane PW (2013). Risky single-occasion drinking and disadvantaged men: will recruitment through primary care miss hazardous drinkers?. Alcohol Clin Exp Res.

[14] Parry O, Bancroft A, Gnich W, Amos A (2001). Nobody home? Issues of respondent recruitment in areas of deprivation. Crit Public Health.

[15] Lindsay J (2005). Getting the numbers: the unacknowledged work in recruiting for survey research. Field Methods.

[16] Kuntsche E, Rehm J, Gmel G (2004). Characteristics of binge drinkers in Europe. Soc Sci Med.

[17] Huckle T, You RQ, Casswell S (2010). Socio-economic status predicts drinking patterns but not alcohol-related consequences independently. Addiction..

[18] Grittner U, Kuntsche S, Gmel G, Bloomfield K (2012). Alcohol consumption and social inequality at the individual and country levels—results from an international study. Eur J Pub Health.

[19] Schrijvers CTM, van de Mheen HD, Stronks K, Mackenbach JP (1998). Socioeconomic inequalities in health in the working population: the contribution of working conditions. Int J Epidemiol.

[20] Collins SE (2016). Associations between socioeconomic factors and alcohol outcomes. Alcohol Res.

[21] Bath & North East Somerset Council. Voicebox 24. 2016.

[22] Office for National Statistics. SOC2010 volume 3: The National Statistics Socio-economic classification (NS-SEC rebased on SOC2010). 2010 [Available from: https://www.ons.gov.uk/methodology/classificationsandstandards/standardoccupationalclassificationsoc/soc2010/soc2010volume3thenationalstatisticssocioeconomicclassificationnssecrebasedonsoc2010.

[23] Bush K, Kivlahan DR, McDonell MB, Fihn SD, Bradley KA (1998). Project ftACQI. The AUDIT alcohol consumption questions (AUDIT-C): an effective brief screening test for problem drinking. Arch Intern Med.

[24] NHS. Binge drinking 2019 [Available from: https://www.nhs.uk/live-well/alcohol-support/binge-drinking-effects/.

[25] Public Health England. Have a Word Scratchcard. 2017 [Available from: https://www.e-lfh.org.uk/wp-content/uploads/2017/12/PHE-Have-a-Word-Scratchcard-Updates-v1_0.pdf.

[26] Elo S, Kyngäs H (2008). The qualitative content analysis process. J Adv Nurs.

[27] Erlingsson C, Brysiewicz P (2017). A hands-on guide to doing content analysis. Afr J Emerg Med.

[28] Public Health England. Local Alcohol Consumption Survey National Report. 2017. Available from: https://assets.publishing.service.gov.uk/government/uploads/system/uploads/attachment_data/file/740438/Local_alcohol_consumption_survey_report.pdf.

[29] Office for National Statistics. Adult drinking habits in Great Britain 2018 [Available from: https://www.ons.gov.uk/peoplepopulationandcommunity/healthandsocialcare/drugusealcoholandsmoking/datasets/adultdrinkinghabits.

[30] McCormack M, Adams A, Anderson E. Taking to the streets: the benefits of spontaneous methodological innovation in participant recruitment. Qual Res. 2012;13(2):228–41. 10.1177/1468794112451038.

[31] Edwards P, Roberts I, Clarke M, DiGuiseppi C, Pratap S, Wentz R, Kwan I (2002). Increasing response rates to postal questionnaires: systematic review. BMJ..

[32] Sauermann H, Roach M (2013). Increasing web survey response rates in innovation research: an experimental study of static and dynamic contact design features. Res Policy.

[33] Porter SR (2004). Raising response rates: what works?. New Dir Inst Res.

[34] Tuten TL, Galesic M, Bosnjak M (2004). Effects of immediate versus delayed notification of prize draw results on response behavior in web surveys: an experiment. Soc Sci Comput Rev.

[35] Bloomfield KIM, Grittner U, Kramer S, Gmel G. Social Inequalities In Alcohol Consumption and Alcohol-related Problems in the Study Countries of the EU Concerted Action ‘Gender, Culture and Alcohol Problems: A Multi-national Study’. Alcohol Alcohol. 2006;41(suppl_1):i26–36. 10.1093/alcalc/agl073. 10.1093/alcalc/agl073 17030500

[36] Roerecke M, Rehm J (2010). Irregular heavy drinking occasions and risk of ischemic heart disease: a systematic review and meta-analysis. Am J Epidemiol.

[37] Sundell L, Salomaa V, Vartiainen E, Poikolainen K, Laatikainen T (2008). Increased stroke risk is related to a binge drinking habit. Stroke..

[38] Kuntsche E, Kuntsche S, Thrul J, Gmel G (2017). Binge drinking: health impact, prevalence, correlates and interventions. Psychol Health.

[39] NHS Digital. Health Survey for England 2019. 2020 [Available from: https://digital.nhs.uk/data-and-information/publications/statistical/health-survey-for-england/2019.

[40] Frone MR, Brown AL (2010). Workplace substance-use norms as predictors of employee substance use and impairment: a survey of U.S. workers. J Stud Alcohol Drugs.

[41] Cooke R, Dahdah M, Norman P, French DP (2016). How well does the theory of planned behaviour predict alcohol consumption? A systematic review and meta-analysis. Health Psychol Rev.

[42] Grzywacz JG, Almeida DM (2008). Stress and binge drinking: a daily process examination of stressor pile-up and socioeconomic status in affect regulation. Int J Stress Manag.

[43] Cooper ML, Russell M, Skinner JB, Frone MR, Mudar P (1992). Stress and alcohol use: moderating effects of gender, coping, and alcohol expectancies. J Abnorm Psychol.

[44] Kelly MP, Barker M (2016). Why is changing health-related behaviour so difficult?. Public Health.

[45] De Visser RO, Birch JD (2012). My cup runneth over: young people's lack of knowledge of low-risk drinking guidelines. Drug Alcohol Rev.

[46] Rosenberg G, Bauld L, Hooper L, Buykx P, Holmes J, Vohra J. New national alcohol guidelines in the UK: public awareness, understanding and behavioural intentions. J Public Health. 2017;40(3):549–56. 10.1093/pubmed/fdx126. 10.1093/pubmed/fdx126 PMC6166584 28977621

[47] Ellard-Gray A, Jeffrey NK, Choubak M, Crann SE. Finding the hidden participant: solutions for recruiting hidden, hard-to-reach, and vulnerable populations. Int J Qual Methods. 2015;14(5):1609406915621420. 10.1177/1609406915621420.

[48] Shavers VL (2007). Measurement of socioeconomic status in health disparities research. J Natl Med Assoc.

